# Remote monitoring titration clinic to implement guideline-directed therapy for heart failure patients with reduced ejection fraction: a pilot quality-improvement intervention

**DOI:** 10.3389/fcvm.2023.1202615

**Published:** 2023-06-19

**Authors:** Erick Romero, Stella Yala, Camryn Sellers-Porter, Genevieve Lynch, Veronicah Mwathi, Yvette Hellier, Svetlana Goldman, Paulo Rocha, Jeffrey R. Fine, David Liem, Julie T. Bidwell, Imo Ebong, Michael Gibson, Martin Cadeiras

**Affiliations:** ^1^ Division of Cardiovascular Medicine, UC Davis Medical Center, Sacramento, CA, United States; ^2^ Department of Public Health Sciences, University of California Davis, Davis, CA, United States; ^3^ Betty Irene Moore School of Nursing, University of California Davis, Sacramento, CA, United States

**Keywords:** titration, GDMT, remote monitoring, HFrEF, implementation, quality of care

## Abstract

**Introduction:**

Guideline-directed medical therapy (GDMT) is the recommended treatment for heart failure with reduced ejection fraction (HFrEF). However, the implementation remains limited, with suboptimal use and dosing. The study aimed to assess the feasibility and effect of a remote monitoring titration program on GDMT implementation.

**Methods:**

HFrEF patients were randomly assigned to receive either usual care or a quality-improvement remote titration with remote monitoring intervention. The intervention group used wireless devices to transmit heart rate, blood pressure, and weight data daily, which were reviewed by physicians and nurses every 2–4 weeks. Medication tolerance was assessed via phone, and dosage instructions were given. This workflow was repeated until target doses were reached or further adjustments were not tolerated. A 4-GDMT score measured use and target dosage, with the primary endpoint being the score at 6 months follow-up.

**Results:**

Baseline characteristics were similar (*n* = 55). A median of 85% of patients complied with transmitting device data every week. At the 6-month follow-up, the intervention group had a 4-GDMT score of 64.6% compared to 56.5% in the usual care group (*p* = 0.01), with a difference of 8.1% (95% CI: 1.7%–14.5%). Similar results were seen at the 12-month follow-up [difference 12.8% (CI: 5.0%–20.6%)]. The intervention group showed a positive trend in ejection fraction and natriuretic peptides, with no significant difference between groups.

**Conclusions:**

The study suggests that a full-scale trial is feasible and that utilizing a remote titration clinic with remote monitoring has the potential to enhance the implementation of guideline-directed therapy for HFrEF.

## Introduction

In patients with heart failure (HF) with reduced ejection fraction (HFrEF), guideline-directed medical therapy (GDMT) is the established treatment to decrease mortality and hospitalization rates (1, 2). Despite the fact that the clinical benefits of GDMT are well established, implementation is poor in clinical practice, with important gaps in use and dosing (3–5). Furthermore, the adoption of novel guideline therapy is also limited (6). These gaps in implementation may be due to various factors, including lack of awareness or knowledge of the guidelines, concerns about potential side effects or adverse events, patient-specific factors such as comorbidities or medication intolerances, and inadequate follow-up or monitoring of therapy (7, 8). However, the reasons for undertreatment of these patients are not well understood and rarely documented (9, 10).

Therefore, quality-improvement strategies are urgently needed to address these gaps in implementing treatment guidelines. Exploring strategies tested in real-life settings can provide valuable insight into what is feasible in routine clinical care and contribute to establishing standard practices. Pragmatic trials are particularly useful for this purpose (11). Close monitoring of vital signs and weight for initiation and titration of GDMT medications is fundamental but difficult to obtain with traditional in-person visits. Telehealth strategies such as noninvasive remote monitoring to capture blood pressure, heart rate, and weight can give clinicians valuable data to guide medication titration (12). A titration program aided by remote monitoring could be a promising approach to improve GDMT use and dosing. To this end, a remote titration clinic program was developed, consisting of nurses, pharmacists, and physicians aided by remote monitoring.

The objective of this pilot study was to evaluate the feasibility and effect of the proposed program on implementing guideline-directed therapy for HFrEF. To evaluate feasibility in real-world clinical care, the study used a pragmatic study design (11) and evaluated remote monitoring compliance, healthcare utilization, GDMT quality of care, and safety measures.

## Methods

### Design

Clinical trials can be traditional or pragmatic or something in between—this is sometimes referred to as the Pragmatic–Explanatory Continuum concept (13). Traditional (or explanatory) trials typically rely on follow-up, additional resources, and healthcare professionals that are beyond standard practice, which may often be unfeasible to implement in routine care. At the other end of the spectrum, a pragmatic trial design allows for flexibility in the delivery of the intervention and titration follow-up to mimic real-world clinical care (11). We aimed to study our proposed quality intervention in a real-world setting that is similar to the one in which the intervention is intended to be implemented as part of normal care in the future (Supplementary Table S1). Specifically, the present study was a pilot pragmatic clinical trial to evaluate the feasibility of a remote titration clinic intervention assisted by remote monitoring vs. usual care only and its impact on GDMT implementation. After 6 months in the usual care group, participants were permitted to crossover to the intervention group at the discretion of the treating physician. The study was institutional review board approved, adhered to the Declaration of Helsinki, and registered on clinicaltrials.gov (NCT04196842).

### Patients

Eligibility included outpatient HFrEF patients with left ventricular ejection fraction (LVEF) ≤40% and taking at least one GDMT medication class at <50% of the target dose. GDMT medication classes considered in this study included angiotensin-converting enzyme inhibitor, angiotensin receptor blocker, or angiotensin receptor neprilysin inhibitor (ACEI/ARB/ARNI), β-blockers (BBs), and mineralocorticoid receptor antagonists (MRAs). Exclusion criteria included comfort care/hospice, waiting for or history of heart transplantation or left ventricular assist device, an estimated glomerular filtration rate of <30 (ml/min/1.73 m^2^), or dialysis. After written informed consent was obtained, a simple randomization method was used to assign participants to either the intervention or usual care group. Participants assigned to the quality-improvement intervention were guided through the process of using these devices by a research coordinator. Devices consisted of a conventional blood pressure monitor and a scale with an embedded wireless data transmission feature (IHealth Inc., CA, United States, or BodyTrace Inc., CA, United States).

### Quality-improvement intervention: implementation of GDMT through a remote titration clinic with remote monitoring

**Figure 1:**
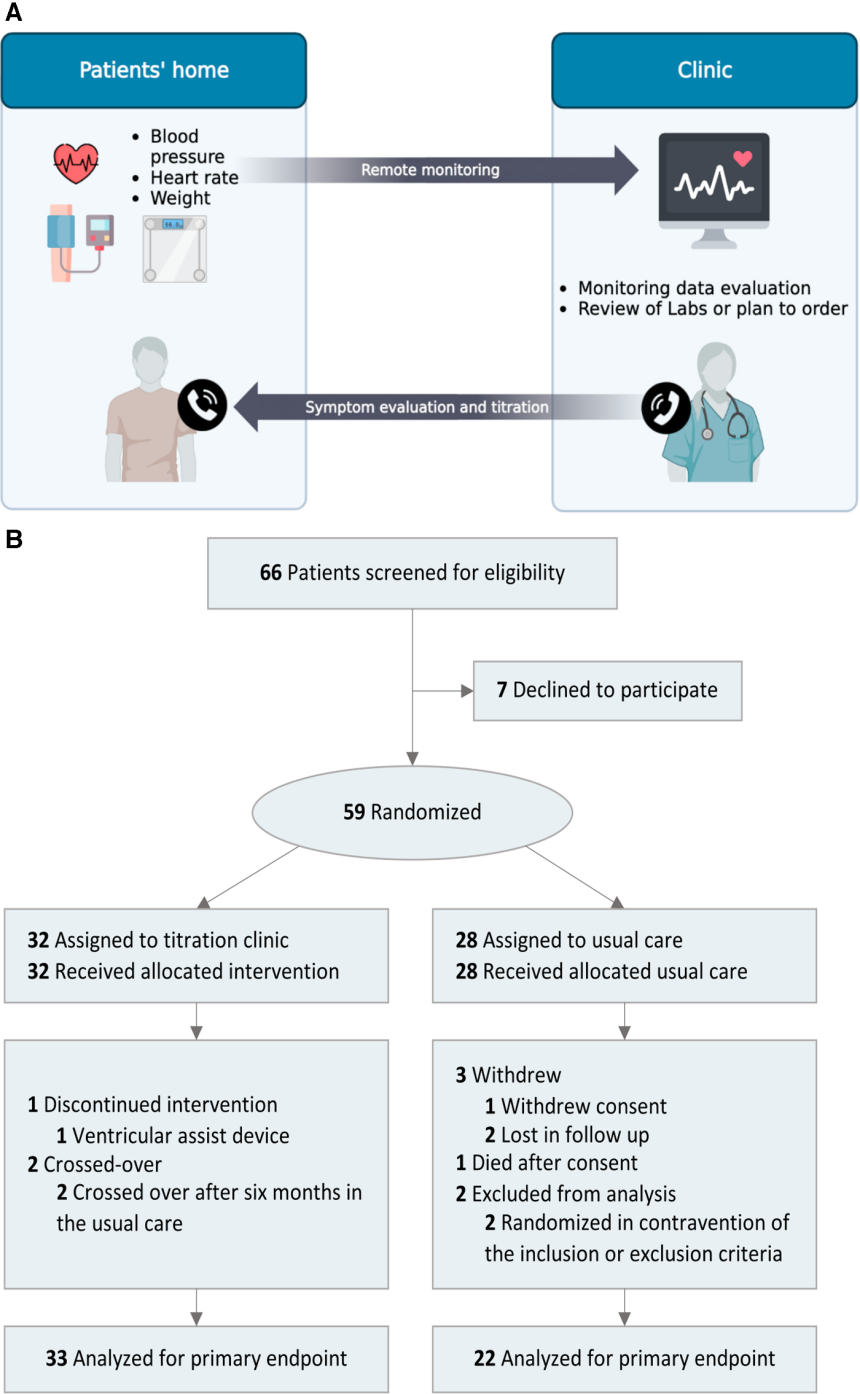
(**A**) Remote titration clinic for GDMT implementation workflow. The remote titration clinic's goal was to implement clinical practice guidelines in a pragmatic design to mimic real-world clinical care. Patient's daily weight, blood pressure, and heart rate were transmitted wirelessly. Healthcare professionals evaluated the monitoring data every 2–4 weeks. Subsequently, medication tolerance (symptoms, vital signs, and lab values) was assessed/discussed via phone, lab tests were ordered if required, and new titration instructions were given. GDMT, guideline-directed medical therapy. (**B**) Flow of patients through the study.

Patients in both groups received the usual care from their treating health providers. The intervention group received the remote titration clinic intervention and usual care. The goal of the titration clinic was to implement HFrEF clinical practice guidelines (1, 2) in a pragmatic design to mimic real-world clinical care, with flexibility in the intervention delivery and titration follow-up (11). Since recommendations supporting sodium-glucose transport protein 2 inhibitors (SGLT2i) were released during the study, we adopted the new recommendations, considering our study's pragmatic design and patient safety (2). From home, patients recorded daily remote monitoring data of weight, blood pressure, and heart rate and wirelessly transmitted the data to a secure data server (Vitally Inc., CA, United States) or to electronic health records. Following the clinical practice guidelines, nurses or physicians reviewed the monitoring data and any available laboratory results every 2–4 weeks. Afterward, patients were contacted via phone to assess medication tolerance, and laboratory tests were ordered by the healthcare provider if considered clinically indicated. Finally, patients were given new dosing instructions in coordination with pharmacists. This workflow was repeated iteratively every 2–4 weeks until patients reached target doses or further dose increases were no longer tolerated (Figure 1A). Symptoms were evaluated alongside other vital signs and laboratory values to assess maximally tolerated GDMT during every remote contact as part of the workflow. The determination of maximally tolerated GDMT was generally dependent on heart rate (≤55 beats/min), symptomatic hypotension (i.e., worsening fatigue or dizziness), blood pressure ≤90 mmHg, serum potassium levels (≥5.5 mEq/L), renal function (creatinine increase to 3.1–3.5 mg/dl), or the need for referral to advanced therapies. We followed medication contraindications as recommended by guidelines (1, 2, 14). Clinical laboratory and echocardiographic assessments were ordered every 3–6 months, although the frequency of these tests could be adjusted based on individual patient requirements or as determined by the remote titration clinician (pragmatic design). Usual care patients had clinical encounters, phone calls, labs, and medication titration, also as determined by the treating clinician.

### Study endpoints and measures

The endpoint measure used a quality-of-care score that evaluated the four GDMT pillars, ACEI/ARB/ARNI, BB, MRA, and SGLT2i. For each medication class, a value of 0 (not treated), 1 (<50% of target dose), or 2 (≥50% of target dose) points was assigned. The maximum points attainable were 8, and the 4-GDMT score was reported as a percentage of the maximum achievable points. The primary endpoint was the 4-GDMT score achieved at the 6-month follow-up. A secondary outcome included the score achieved at the 12-month follow-up. Safety and measures included mortality, emergency department visit, HF hospitalizations, symptomatic hypotension, worsening renal function, and hyperkalemia. Healthcare utilization was evaluated as the number of encounters. Exploratory measures included LVEF and natriuretic peptides.

### Statistical analyses

Differences for continuous variables were assessed using Student’s *t*-test or Wilcoxon signed-rank test depending on the distribution of the data. For categorical variables, Fisher's exact or *χ*^2^ test was used as appropriate. The simple randomization was carried out using a computer-generated sequence to generate random numbers using SAS software. The primary and secondary endpoint analyses were performed using analysis of covariance (ANCOVA) models adjusted by baseline as a covariate and Bonferroni correction. The brain natriuretic peptide (BNP) and N-terminal proBNP (NT-proBNP) values were log-transformed for analysis. Linear mixed models were used to analyze longitudinal LVEF, BNP, and NT-proBNP. Analyses were performed using SAS software version 9.4 (SAS Institute, Cary, NC, United States).

## Results

### Study participants and remote monitoring compliance

Patients were enrolled from the outpatient HF clinic at the University of California, Davis (UCD) medical center between 2020 and 2021. The study had an 89% consent rate; the flow of patients through the study is depicted in Figure 1B. Regarding compliance, a median (IQR) of 85% (82%–88%) of patients transmitted remote monitoring data every week. Five participants encountered technical difficulties due to device handling and usage by; all were resolved through troubleshooting and support. A total of 55 patients were included in the final analysis. The mean patient age was 62 years with a high comorbidity burden. Overall, baseline characteristics were similar (Table 1), except for implantable cardiac defibrillators.

**Table 1:** Baseline characteristics.

	Titration clinic (*n* = 33)	Usual care (*n* = 22)	*p*-value
Age (years), mean (±SD)	61.3 (12.3)	63.5 (11.3)	0.40
Male, no. (%)	21 (64)	16 (74)	0.48
Race, no. (%)			0.20
White	19 (58)	15 (68)	
Black	2 (6)	5 (22)	
Asian	4 (12)	1 (5)	
Other/unknown	8 (24)	1 (5)	
Ethnicity, no. (%)			0.13
Hispanic	7 (21)	1 (5)	
Non-Hispanic	26 (79)	21 (95)	
NYHA functional classification, no. (%)			0.20
II	19 (58)	8 (36)	
III	14 (42)	13 (59)	
AHA classification, no. (%)			1.00
C	32 (97)	21 (95)	
D	1 (3)	1 (5)	
Systolic blood pressure (mm Hg), mean (±SD)	111.6 (13.7)	115.4 (12.5)	0.30
Diastolic blood pressure (mm Hg), mean (±SD)	68 (10.8)	70.9 (7.1)	0.26
Heart rate (bpm), mean (±SD)	77.9 (12.3)	76.9 (14.2)	0.78
Body mass index, mean (±SD)	30.5 (8.1)	29.4 (4.5)	0.53
Sodium (mEq/L), mean (±SD)	137.9 (2.6)	137.5 (1.9)	0.48
Potassium (mEq/L), mean (±SD)	4.1 (0.3)	4.1 (0.4)	0.70
Blood urea nitrogen (mg/dl), mean (±SD)	22.4 (8.4)	25.1 (10.8)	0.30
Creatinine (mg/dl), mean (±SD)	1.2 (0.4)	1.4 (0.5)	0.16
Estimated glomerular filtration rate (ml/min/1.73 m^2^), mean (±SD)	64.8 (18.7)	58 (20.6)	0.21
Left ventricular ejection fraction %, median (IQR)	25 (20–30)	30 (18–35)	0.39
Brain natriuretic peptide, median (IQR)	447 (197.5–1,394)	264 (199–544)	0.21
N-terminal ProBNP, median (IQR)	1,044 (191–1,849)	922 (312–2,027)	0.64
Medical history, no. (%)			
Implantable cardioverter-defibrillators	16 (49)	5 (23)	0.05
Coronary artery disease	14 (42)	11 (50)	0.58
Hyperlipidemia	17 (52)	8.0 (36.36)	0.27
Hypertension	21 (64)	15.0 (68.18)	0.73
Diabetes	11 (33)	10 (45)	0.36
Atrial fibrillation	18 (55)	16 (73)	0.17
Valvular disease	18 (55)	11 (50)	0.74
Pulmonary hypertension^a^	2 (6)	3 (14)	0.37
Chronic kidney disease	14 (42)	10 (46)	0.82
Asthma/COPD/OSA	11 (33)	3 (14)	0.10
History of cancer	5 (15)	2.0 (9)	0.50
Baseline medication use, no. (%)			
ACEI, ARB, or ARNI	31 (94)	17 (77)	0.10
β-blocker	33 (100)	21 (95)	0.40
Mineralocorticoid receptor antagonist	23 (70)	16 (73)	0.80
Sodium-glucose transport protein 2 inhibitors	0	1 (5)	0.40

ACEI, angiotensin-converting enzyme inhibitor; AHA, American Heart Association; ARB, angiotensin receptor blocker; ARNI, angiotensin receptor neprilysin inhibitor; BB, β-blocker; COPD, Chronic obstructive pulmonary disease; MRA, mineralocorticoid receptor antagonist; NYHA, New York Heart Association; OSA, Obstructive sleep apnea.

^a^ Other than Group 1 pulmonary arterial hypertension.

### Endpoints

**Figure 2:**
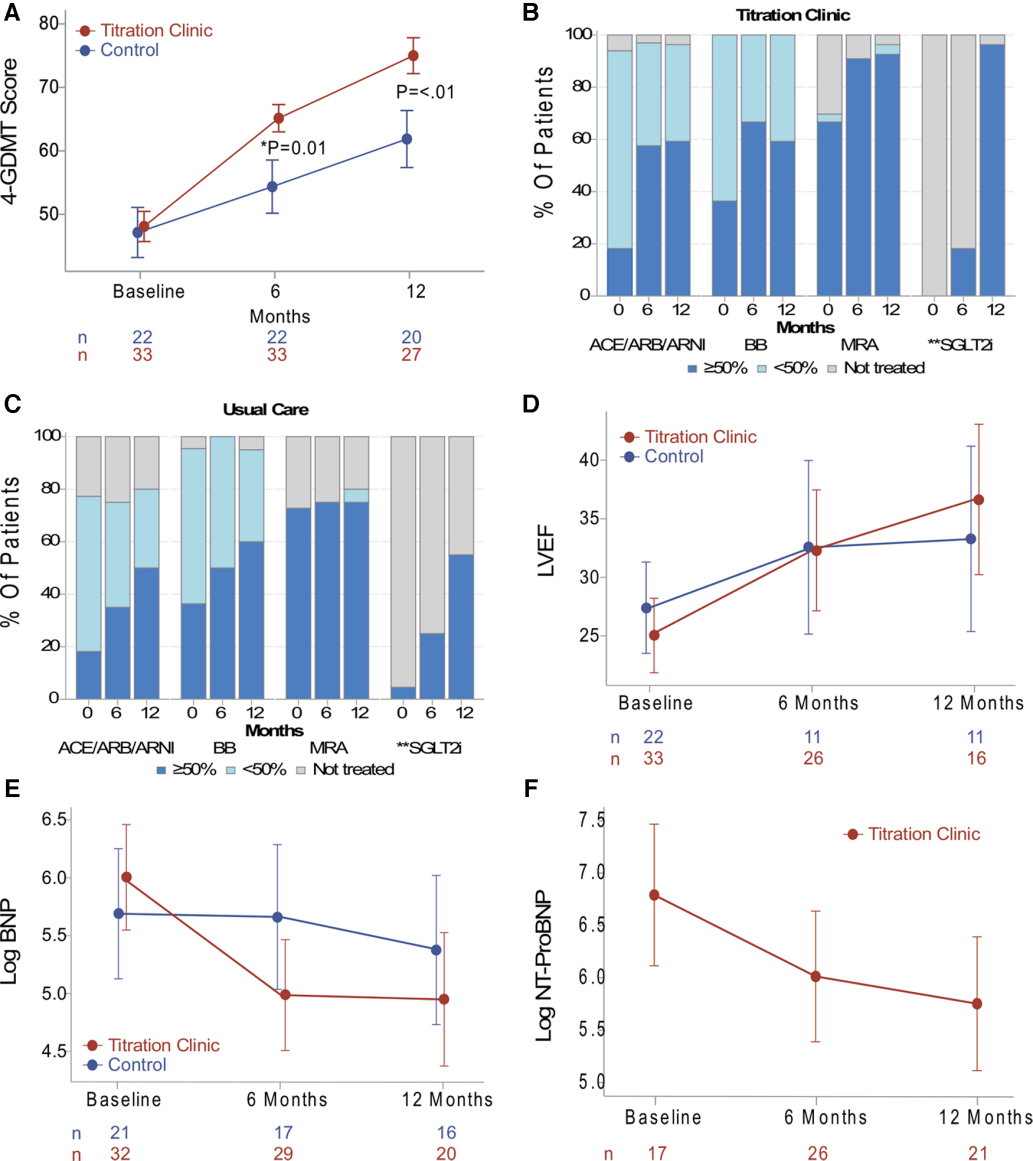
(**A**) data presented as the absolute mean and error bars indicating standard errors. The primary endpoint was the GDMT score at 6 months, and the titration clinic group had a higher score compared to usual care (*p *= 0.01). The secondary endpoint was at 12 months, and the titration clinic group remained higher than usual care (*p *< 0.01). (**B, C**) Patient percent of GDMT use and target dose for both study groups. (**D**) Data on the trend of LVEF, (**E**) BNP, and (**E**) NT-proBNP presented as least-squares means and error bars indicating confidence intervals derived from linear mixed models. There were no significant differences between the groups over time. However, the titration clinic group showed a positive LVEF, BNP, and NT-proBNP trend in a *post-hoc* within-group comparison (*p *< 0.05). *Primary endpoint. **Medication class categorized as treated (dark blue) or not treated (gray). GDMT, guideline-directed medical therapy; ACEI, angiotensin-converting enzyme inhibitor; ARB, angiotensin receptor blocker; ARNI, angiotensin receptor neprilysin inhibitor; ANCOVA, analysis of covariance; BB, β-blocker; BNP; brain natriuretic peptide; CI, confidence interval; Log, logarithm transformation; LVEF, left ventricular ejection fraction; MRA, mineralocorticoid receptor antagonist; NT-proBNP, N-terminal proBNP.

At the primary endpoint, at the 6-month follow-up, the intervention group showed an increase in the 4-GDMT quality score of 64.6% vs. 56.5% (*p *= 0.01) in the usual care group; difference of 8.1% (95% CI: 1.7%–14.5%). At the secondary endpoint, at 12 months, there was a score increase in both groups. However, the intervention group had a higher GDMT score compared to usual care, 74.9% vs. 62.1%, respectively (*p *= <.01), a difference of 12.8% (95% CI: 5.0%–20.6%). The results are summarized in Figure 2A. Absolute target dose changes over time are depicted in Figures 2B, C. In an intention to treat analysis for the primary endpoint, differences retained statistical significance (*p *= 0.043) (Supplementary Table S2).

### Safety and healthcare utilization measures

A set of safety measures was assessed, with no significant differences (Table 2). Symptomatic hypotension was relatively common in both groups (titration clinic 27% and usual care 23%). Worsening renal function and hyperkalemia occurred in ≤6% of patients. Mortality, hospitalizations, and emergency room visits were similar among the study groups, and no major adverse events were reported. In terms of healthcare utilization, as anticipated, the titration clinic involved regular remote encounters, in line with the treatment guidelines (1, 2). This resulted in a higher number of encounters, indicating improved implementation of the treatment guidelines; however, additional resources are needed to implement treatment guidelines.

**Table 2:** Safety and healthcare utilization measures.

	Titration clinic	Usual care	*p*-value
Mortality, *n* (%)	0	1 (5)	0.40
Left ventricular assist device, *n* (%)	1 (3)	0	1.00
HF hospitalizations, *n* (%)	7 (21)	4 (18)	0.72
Cardiovascular ED visits, *n* (%)^a^	5 (15)	2 (9)	0.68
Symptomatic hypotension, systolic ≤90 mmHg, *n* (%)	8 (24)	5 (23)	0.76
Worsening renal function, Cr > 3.1–3.5, *n* (%)	2 (6)	1 (5)	1.00
Hyperkalemia, K > 5.5, *n* (%)	1	0	1.00
Implantable cardioverter-defibrillators, *n* (%)	3 (9)	1 (5)	0.64
CardioMEMS, *n* (%)	2 (6)	0	0.51
Procedures, mitraclip or ablation, *n* (%)	2 (6)	2 (9)	1.00
Number of encounters^b^, median (IQR)	12 (9–16)	4 (3–6)	<0.01

ED, emergency department; HF, heart failure; Cr, creatinine; K, potassium.

Analysis was performed using Fisher's exact test and Student's *t*-test.

^a^ The number of ED visits related to medication titration was 1 (3%) in the intervention and 1 (5%) in the usual care group.

^b^ The number of encounters includes usual care and the remote titration clinic intervention.

### Exploratory measures

The time course of changes in LVEF, BNP, and NT-proBNP levels is depicted in Figures 2D–F, respectively. Linear mixed models resulted in no significant differences between groups over time. However, in a *post-hoc* within-group comparison, the titration clinic intervention group showed a positive trend in ejection fractions and natriuretic peptides (*p* < 0.05). Of note, laboratory tests were determined by the treating clinicians in both groups, with NT-proBNP less commonly ordered for patients in the usual care group. Thus, the NT-proBNP analysis was performed for the remote titration group only. Additionally, there was a decreasing trend over time of blood pressure and heart rate, which may reflect the uptitration of GDMT medications (Supplementary Figure S1).

## Discussion

This pragmatic pilot study was conducted to determine the feasibility of a remote titration clinic assisted with remote monitoring and to assess the impact on the implementation of GDMT. Patient compliance with remote monitoring was relatively high (85%). A small proportion of patients experienced technical issues that were resolved. Findings suggest that the proposed approach can enhance GDMT use and attainment of target doses in clinical practice. We observed that at 6 months (primary endpoint), the remote titration group had an improvement in the use and dose as measured by the 4-GDMT quality score. This improvement pattern was sustained through the 12-month study period. Interestingly, the target dose of BB was reduced at the 12-month follow-up in the remote titration group (Figure 2B). This may be due to the initiation and/or uptitration of the other medication classes. However, specific factors for dose reductions at the drug class level were not assessed. The frequent remote titration encounters in the intervention group, which were in accordance with the treatment guidelines (1, 2), suggest an improved implementation of the clinical practice guidelines compared to usual care. This also informs about the resources needed to implement the treatment guidelines.

Echocardiographic and biomarker analyses in this study did not yield significant differences between the two groups over time. However, a positive trend in LVEF and a decrease in natriuretic peptides in the remote titration group were found in a *post-hoc* within-group analysis. No severe adverse events were documented, and the incidence of intolerances, such as symptomatic hypotension, worsening renal function, and hyperkalemia, were similar for both groups.

Previous studies to improve GDMT implementation have been proposed. In the outpatient setting, medication use and dose improved in a nurse-directed titration program (15). Similarly, an algorithm-guided program directed by health navigators and pharmacists resulted in increased use of ACEI/ARB/ARNI and BB (16). In the inpatient setting, patients with noncardiovascular hospitalizations had an improvement in GDMT at discharge and 30-day follow-up with a virtual GDMT team (17). Taken together, these studies suggest that systematic uptitration is possible, further supporting our findings. Key differences between previous studies and the present study include remote monitoring, randomization design, and an evaluation of HF clinical measures such as LVEF and natriuretic peptides. In addition, our pragmatic design provides valuable insights into what can be accomplished in routine clinical care, which may ultimately support adoption into clinical practice.

This study had limitations. First, the sample size was relatively small. Despite this, we retained adequate power to test the primary endpoint. Second, this study was carried out in an outpatient HF clinic of a single academic center, and the results may not be generalizable to other settings. Third, the intervention was not blinded, as the overt intervention did not allow for the blinding of the investigators or patients. Fourth, while compliance with remote monitoring was acceptable, patient self-reported acceptability or satisfaction was not collected. We also did not collect data on family caregiver presence or whether/how they may have facilitated remote monitoring in the home. Patient- and caregiver-reported outcomes measures, acceptability, and blinding strategies should be addressed in future studies. Fifth, the COVID-19 pandemic slowed enrollment in both study groups, resulting in a delay in study completion.

## Conclusions

This study suggests that a remote titration program assisted by remote monitoring is feasible and has the potential to enhance the implementation of HFrEF treatment guidelines. A larger study is needed to generate more robust evidence of comparative effects.

## Data Availability

The raw data supporting the conclusions of this article will be made available by the authors upon request.
