# Supplementary material for: Remote monitoring titration clinic to implement guideline-directed therapy for heart failure patients with reduced ejection fraction: a pilot quality-improvement intervention
Source: Front Cardiovasc Med. 2023 Jun 19;10:1202615. doi: 10.3389/fcvm.2023.1202615 (PMC10316022; doi:10.3389/fcvm.2023.1202615)
Supplement: Supplementary file 1 [file Datasheet1.docx]

**Supplementary material**

| **Table S1:** PRECIS-2 tool scores for trial domains | | | |
| --- | --- | --- | --- |
|  | **Domain** | **Score** | **Rationale** |
| **1** | **Eligibility Criteria** | 5 | Participants in the trial are similar to those who would receive this intervention if it was part of usual care |
| **2** | **Recruitment Path** | 5 | Recruitment through usual appointments or clinic |
| **3** | **Setting** | 3 | The study was conducted in a single center, specialized heart failure and academic center |
| **4** | **Organisation intervention** | 3 | Require more than usual, and increase resources, but not completely different. The extra effort is in organization |
| **5** | **Flex of experimental intervention – Delivery** | 5 | Very pragmatic choice with identical flexibility to usual care |
| **6** | **Flex of experimental intervention – Adherence** | 4 | More than usual encouragement to adhere to the intervention. However, this is not an exclusion, rater is part of the study intervention |
| **7** | **Follow up** | 4 | More than usual follow up is encouraged. However, this is not an exclusion, rater is part of the study intervention |
| **8** | **Outcome** | 4 | The outcome is of obvious importance to participants and doctors |
| **9** | **Analysis** | 4 | Patients lost in follow up were excluded from the analysis, but a intention to treat analysis was also conducted |
| Tool score domains from the PRECIS-2 tool (Loudon K, et al. BMJ. 2015).  Score: 1, Very explanatory; 2, Rather explanatory; 3, Equally pragmatic/explanatory; 4, Rather pragmatic; 5, Very pragmatic. | | | |

| **Table S2.** Intention to treat analysis for the primary outcome of 4-GDMT score at 6 months | | | | | |
| --- | --- | --- | --- | --- | --- |
| **Parameter** | **Estimate** | **Standard Error** | **LCL** | **UCL** | ***p* -value** |
| Intercept | 56.63 | 2.28 | 52.15 | 61.12 | <.001 |
| Titration Clinic | 8.21 | 2.96 | 2.41 | 14.01 | 0.006 |
| Usual Care | 0 | . | . | . | . |
| Baseline | 0.80 | 0.14 | 0.52 | 1.08 | <.001 |
| Baseline* Titration Clinic | -0.41 | 0.20 | -0.80 | -0.01 | 0.043 |
| Baseline*Usual care | 0 | . | . | . | . |
| The primary endpoint had a significant difference (p=0.043) between the titration clinic and the usual care group. The intention to treat analysis was performed using multiple imputation (*m*=20) and ANCOVA adjusted by baseline as a covariate and Bonferroni correction. All subject randomized to study groups regardless of missing data, lost in follow up or exclusion after randomization were included in the analysis (titration clinic n=32 and usual care n=28). Abbreviations: ANCOVA, analysis of covariance; LCL, lower confidence limits; UCL, upper confidence limits. | | | | | |

**Figure S1.** Heart rate and blood pressure for the remote titration clinic with remote monitoring. Y-axis represents the data value and X-axis represents time in weeks for visualization purposes (data was generated and transmitted daily).
